# Thermal implant removal in a pig jaw: a proof of concept study

**DOI:** 10.1007/s00784-024-05619-0

**Published:** 2024-06-28

**Authors:** Kristian Kniha, Lorenz Haas, Faruk Al-Sibai, Reinhold Kneer, Stephan Christian Möhlhenrich, Benita Hermanns-Sachweh, Frank Hölzle, Ali Modabber

**Affiliations:** 1 Department of Oral and Cranio-Maxillofacial Surgery, University Hospital RWTH, Pauwelstraße 30, Aachen, Germany; 2 Private Clinic for Oral Surgery, Dres, Kniha, Rosental 6, 80331 Munich, Germany; 3 Institute of Heat and Mass Transfer, RWTH Aachen University, Augustinerbach 6, 52056 Aachen, Germany; 4 Department of Orthodontics, University of Witten/Herdecke, Alfred-Herrhausen Str. 45, 58455 Witten, Germany; 5 Private Institute for Implant Pathology, ZBMT, Campus Melaten, Pauwelsstaße 17, Aachen, Germany

**Keywords:** Implant removal, Osteonecrosis, Temperature, Histopathology, Animal model

## Abstract

**Objectives:**

The aim of this study was to evaluate whether thermal implant removal of osseointegrated implants is possible using a diode laser with an specific temperature–time interval.

**Materials and methods:**

First, tooth extraction of the first three premolars was performed in the maxilla and mandible on both sides of 10 pig. After 3 months, implants were inserted into the upper and lower jaws of 10 pigs. After 3 more months, osseointegrated implants were heated with a laser device to a temperature of 50 °C for 1 min. After 14 days, the implant stability quotient (ISQ), torque-out values, and bone-to-implant contact (BIC) ratio were assessed using resonance frequency analysis.

**Results:**

ISQ values showed no significant differences within each group or between the control and test groups. Furthermore, torque-out and BIC value measurements presented no significant differences between the groups.

**Conclusions:**

At 50°C, changes in the BIC values were noticeably smaller; however, these differences were not significant. Future studies should evaluate the same procedures at either a higher temperature or longer intervals.

**Clinical relevance:**

With only 50 °C for 1 min, a dental implant will not de-integrate predictably.

## Introduction

Dental implants have been a common therapeutic procedure to compensate for tooth loss since the 1970s [1]. Since then, pure titanium (99.7%) has prevailed as the main dental implant material. The survival rate of these titanium implants according to the Brånemark protocol was determined to be 90% over a follow-up of 10 years [2].

Therapeutic goals in the context of implant placement include mechanical function (i.e., the ability to chew and speak), tissue health (e.g., osseointegration, preservation of surrounding bone, or freedom from inflammation), and psychology (e.g., freedom from pain or aesthetic appeal) [3, 4].

To provide a functional basis for dental implant loading, the osseointegration of the implant into the bone had to occur. The term osseointegration is defined as the structural and functional connection between the bone and the surface of a load-bearing implant. In such cases, there is no detectable movement of the implant relative to the bone, but there is anchorage of the foreign body material within the vital bone, with no mobility of the two components relative to each other [1, 5, 6]. In this context, the contact surface between the implant and bone is not a homogeneous zone, but instead one that has mineralized, partially mineralized, and completely unmineralized areas [7]. The non-collagenous proteins osteopontin (OPN) and bone sialoprotein (BSP) are arguably instrumental in establishing BIC [8, 9]. Both are thought to be capable of cell adhesion and mineral formation in the area of the BIC [10–12].

However, implantation is not always successful. As with other therapeutic interventions, complications can occur that ultimately result in the removal of the implant. These failures can be temporally divided into early and late variations [13–15]. Implants may need retrieval when they present severe malposition that impede their restoration, when the implant is damaged or fractured, or in the presence of bleeding disorders that preclude traumatic procedures. There are a variety of therapeutic procedures available for the removal of late implant failures [16]. It was shown that by heating the implant surface and the associated reduction in bone contact, it was possible to gently remove the implant.

Case studies have described the use of electrosurgical devices [17, 18], as well as lasers [19], as a source of heat. However, to make such a procedure as reproducible and predictable as possible, certain parameters, such as temperature and duration of the thermal effect, must be established [20]. In this regard, the current body of studies does not provide a clear threshold for the occurrence of bone necrosis [20]. Recent animal studies have evaluated different parameters to break the implant osseointegration by thermal attack. First, a porcine model was used to assess the effects of temperature on bone tissue in vitro [21]. The authors showed that osteocytes died after exposure to 49 °C for 10 s or 5 °C for 30 s. Next to warm and cold temperatures, different temperature devices were analyzed. In another in vitro study, the objective was to empirically find sources that would work well for a controlled implant heating process to produce a uniform temperature distribution threshold [22]. The investigation revealed that clinical applications may be ideal for the thermal distributions of water and laser sources. Additionally, an in vivo investigation of the rat tibia was performed with possible cold and warm temperature–time intervals to determine their effects on non-osseointegrated implants. The authors proposed that a lower bone-to-implant contact (BIC) ratio surrounding implants results from an optimum temperature–time interval for warm and cold temperatures [23]. The results showed that there was a significant reduction in the BIC value at a temperature of 50 °C for 1 min. Thermal necrosis for dental implant removal could be the gentlest method compared to existing treatments. However, no osseointegrated implants were examined in the pilot study. In this study, thermal implant removal at a temperature of 50 °C for 1 min in a pig jaw with osseointegrated implants was performed. The primary aim was the evaluation of changes of the implant stability evaluated with the ISQ, the BIC ratio, and the removal torque of implants treated with thermal shock.

## Methods

### Experimental protocol

**Fig. 1:**
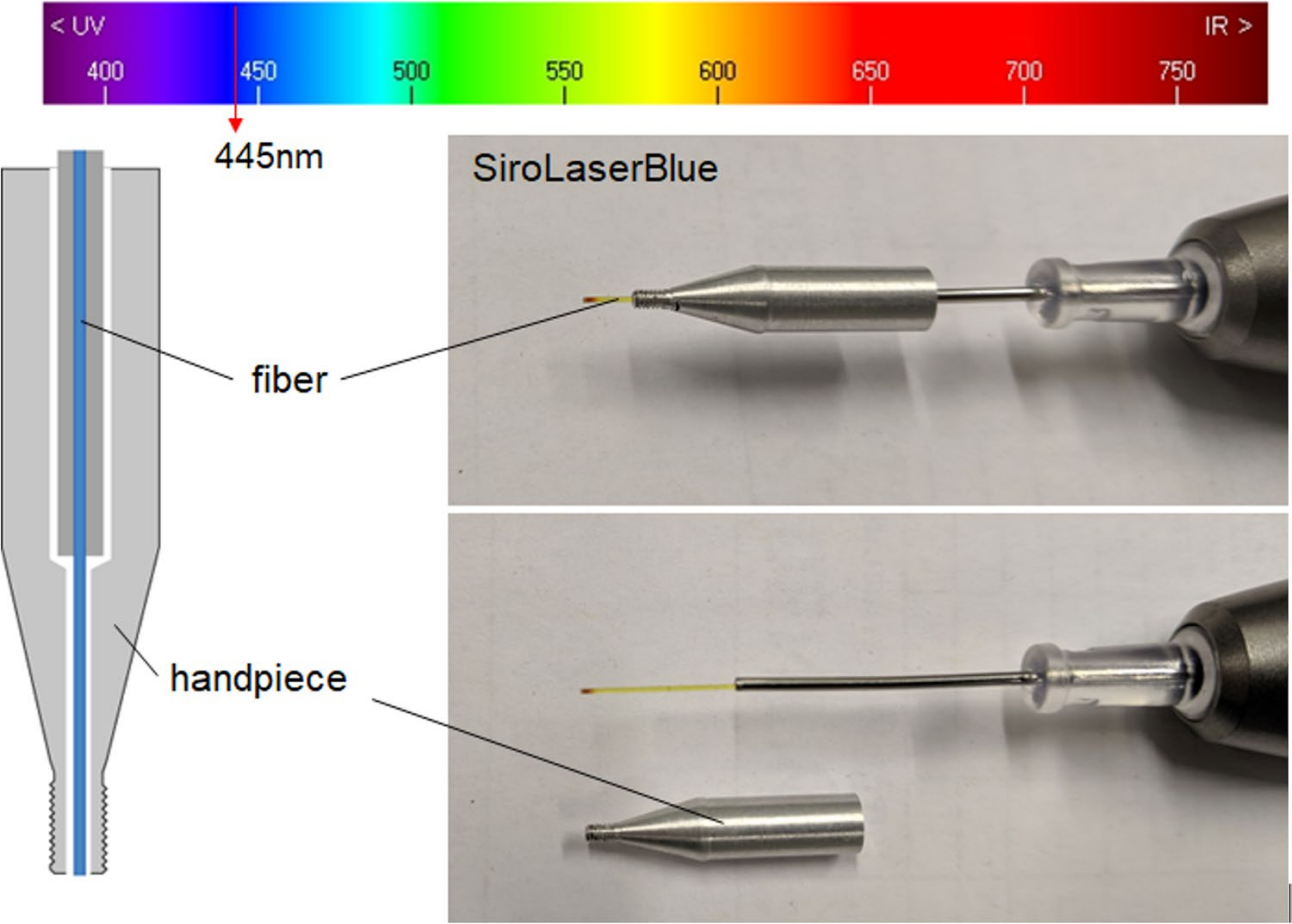
Tempering unit with the diode dental laser “SiroLaser Blue”

In this study, a dental laser was used as a tempering device. The “SiroLaser Blue” dental laser (Dentsply Sirona, Bensheim, Germany) was used for laser heating, in which the laser light (wavelength 445 nm) is emitted through a thin fiber at the end of the handpiece (Fig. 1). The power of the laser ranges from 0.2 to 3.0 W and can be adjusted in 10 mW steps using the pulse mode.

At the beginning of the study, 10 female pigs, each weighing 40–60 kg, were included (adult female Aachener Minipigs, Gerd Heinrichs, Tichelkamp 14, 52525 Heinsberg, Germany). One examiner performed the individual experimental steps of the study. This investigation was carried out in accordance with the guidelines of the European Parliament and the council on the protection of animals used for scientific purposes, Animal Research: Reporting of In Vivo Experiments (ARRIVE) [24] and Directive 2010/63/EU. We confirm that the experimental protocol was approved by a named institutional and licensing committee (Landesamt für Natur und Verbraucherschutz, Recklinghausen, Germany; Ref. 2019A276).

### Tooth extraction – First surgery

All surgical procedures were performed under general anesthesia. Seven days before each operation, the animals were accustomed to a wet food diet. General anesthesia included a premedication with 1 ml/animal 1% atropinsul-fate (10 mg/ml Atropin®)/animal and 0.1–0.2 ml/kg bw Stresnil® [4–8 mg/kg bw Azaperone]) intramuscularly. After 15 min, 0.1 ml/kg (10 mg/kg bodyweight) ketamine at 10% (100 mg/ml Ketanest®) was injected intramuscularly, and after another 10 min, an intravenous line (18G Braunüle®) was inserted into the ear vein. Through this access, the animal received 1 mg/kg (0.1 ml/kg bodyweight) Propofol® 1% (10 mg/ml) for subsequent intubation. After endotracheal intubation, anesthesia was continued with an isoflurane (1.5% by volume)-oxygen (30%) air mixture and fentanyl (6–8-10 µg/h/kg bw; 0.12–0.16–0.2 ml/h/kg bw) via a syringe pump (perfusor). Additionally, a single shot antibiosis (750 mg cefuroxime and 250 mg metronidazole/animal i.v.) was used.

**Fig. 2:**
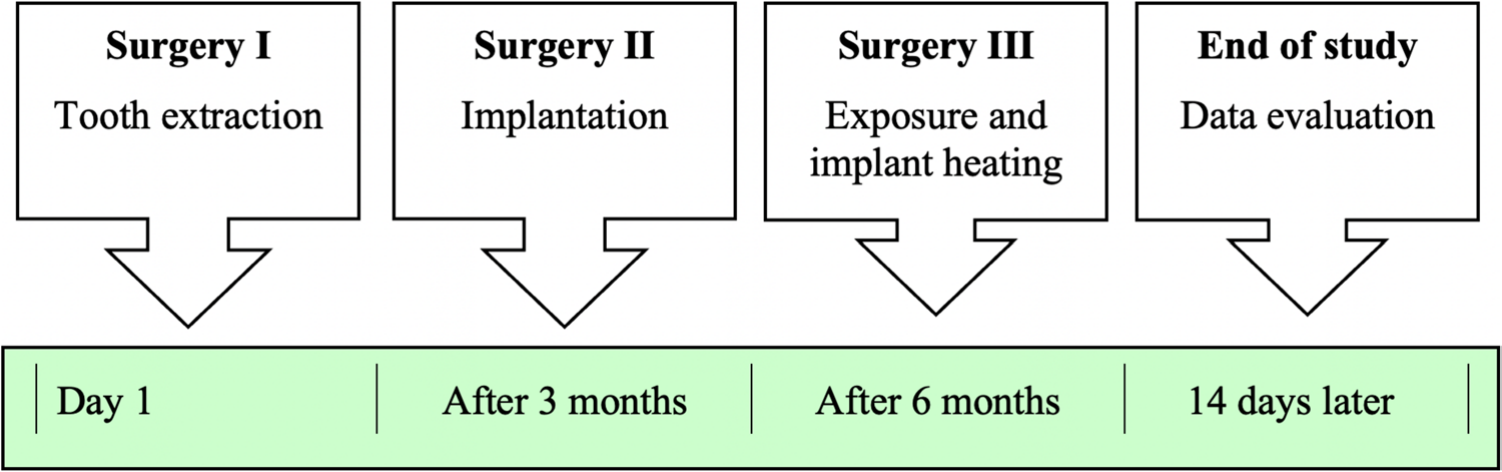
Timeline of the animal study

First, tooth extraction of the first three premolars was performed in the maxilla and mandible bilaterally (Fig. 2). The wounds were covered with a mucoperiosteal flap and resorbable sutures. After each surgery, wet food was fed for 7 days. This surgery was followed by a bony healing phase of 3 months.

### Implant placement – Second surgery

**Fig. 3:**
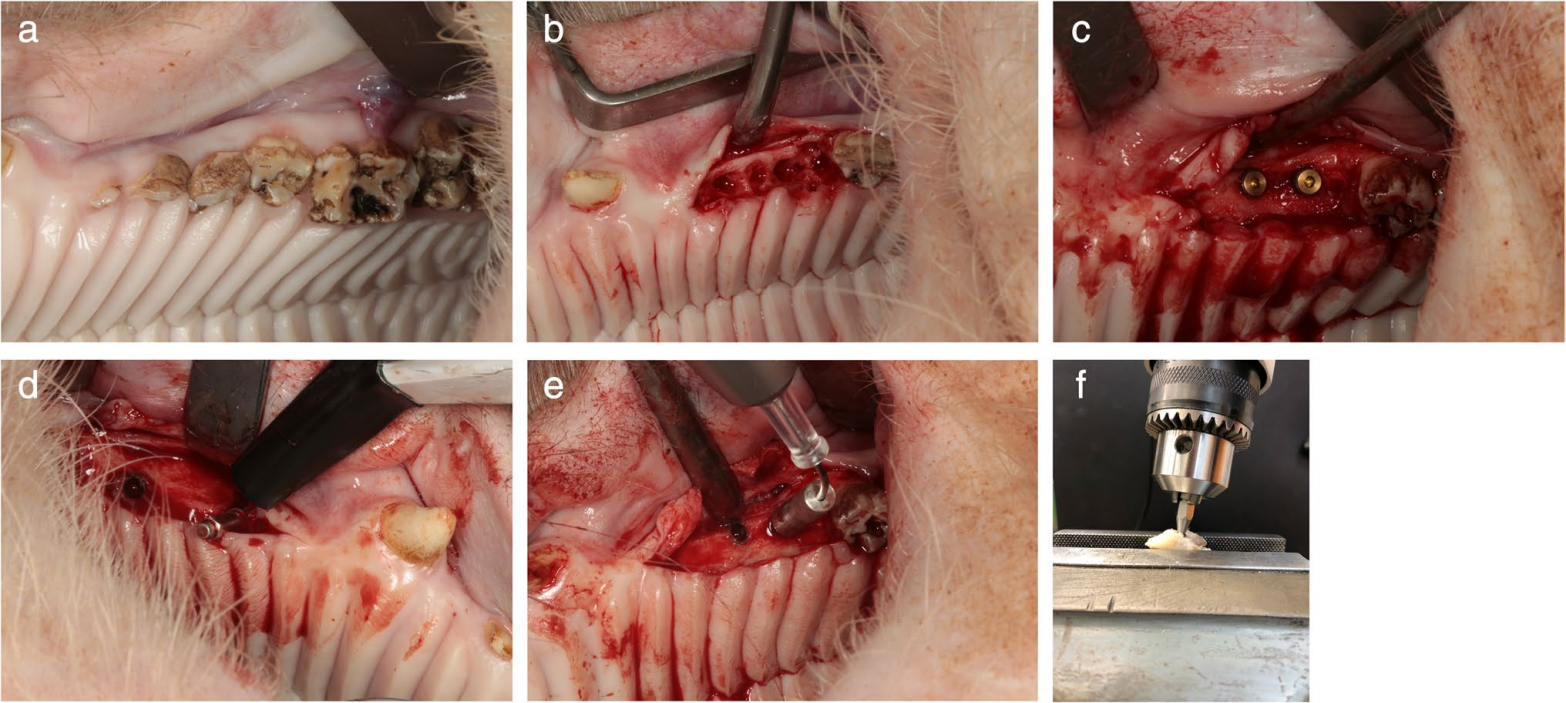
**A **In the first step, tooth extraction of the first three premolars was performed in the maxilla and mandible on both sides. **B **After atraumatic removal, the wounds were closed with sutures. **C **After 3 months, 8 dental implants were placed in the upper and lower jaws (2 implants on each side of each jaw). **D **Implants were inspected, and ISQ values were measured at three different time points. **E **Implants were randomly selected and heated with the laser device at a temperature of 50 °C for 1 min. **F **In the next step, jaw segments were prepared, and the torque-out values for each implant were measured

Two implants (Camlog, screw-line 3.8 × 9 mm titanium implant, Basel, Switzerland) were inserted in each upper and lower hemimandible “ for a total of eight implants per animal. Implantation was performed after exposure of the jawbone with strict cooling with sterile saline. After placing a punch mark, the 2 mm diameter pilot drill was used (drill speed and placement as recommended by the manufacturer). After using the form drill of 3.3 mm diameter, the threat cutting drill was inserted. (Camlog Biotechnologies AG, Basel, Switzerland, Fig. 3). The implant was last inserted with the hand ratchet. Using resonance frequency analysis with hand-screwed individual smart pegs (Osstell, Gothenburg, Sweden), primary stability was evaluated after insertion of the implant. Primary stability was measured with the ISQ value in four directions (i.e., from left to right and from front to back [25]), resulting in a calculated mean ISQ value. After a primary stable insertion of the implants and the healing screw, the wounds were closed with resorbable suture material. This surgery was followed by a bony healing phase of 3 more months.

### Implant exposure and thermal treatment – Third surgery

After the healing phase, the implants were uncovered and inspected, and ISQ values were measured. In the case of implant mobility, implant loss, signs of infection (e.g., bone loss in a dental X-ray film, swelling, pus, or bleeding), and ISQ values < 40, the implant was excluded for thermal removal. Implants were randomly selected and heated with the laser device at a temperature of 50 °C for 1 min. An individual healing cap was used to keep the laser tip in the right place. The laser was set at 1.0 W (pulse mode) for 28 s to heat up the implant surface up to 50 °C. The laser tip was placed into the application device, to point the tip directly into the middle of the implant (Figs. 3 and 7). Then the setting was changed to 0.3W (pulse mode) for 60 s to maintain the temperature. The exact setting were calculated in a vitro set-up simulating the vital pig jaw, which has been used and published before [21, 22]. With the realistic pre-test, the setting and the associated final temperature of the implant surface could be exactly transferred to the animal study. After the insertion of the standard healing cap, the wounds were closed with resorbable suture material. For 14 more days, the animals were kept alive.

### End of the study – Euthanasia

After 14 days, and after sedation and premedication, euthanasia was performed with i.v. administration of a lethal dose of 160 mg/kg body weight (1.0 ml/kg body weight) pentobarbital sodium (Narcoren®, Fa. Rhône Me-rieux, Laupheim). The 14-day follow-up applied here was based on a successful loosening of the implant in another publication [26, 27]. The immediate onset of death was indicated by asystole, maximal mydriasis without light reaction, and extinction of the corneal reflex. This was followed by dissection.

### ISQ and torque-out values and histomorphometric analyses

After ensuring death, the next step was to measure the ISQ values of the implants. After that, the jaw segments were prepared, and the torque-out values for each implant were measured. In the case of a torque value of > 110 Ncm, the implant was rated as still integrated. To avoid damage at the sample we did not apply more than 110 Ncm.

The jaw samples were stored in 4% formalin (neutrally buffered with methanol) for 48 h (Otto Fischar GmbH & Co. KG, Saarbrücken, Germany). The samples were dehydrated using ascending ethanol gradients (50–100%) prior to embedding them in methylmethacrylate resin (Technovit 9100, Heraeus Kulzer GmbH, Frankfurt, Germany). Coronal sections of the embedded undecalcified specimens were obtained at a thickness of approximately 200 μm using an EXAKT cutting unit (EXAKT Technologies Inc., Oklahoma City, Oklahoma, USA). The sections were then thinned and polished manually to a final thickness of about 50–70 µm [28]. Final specimens were stained with toluidine blue according to the protocol and analyzed using light microscopy. One slide for each implant was obtained in the coronal section through the implant center.

The tissue structures were analyzed by one specialist pathologist using digital microscopy. The bone-to-implant ratio was calculated by measuring (μm) the complete circumference in the sectioning of the implant and then recording the area with histologic bone contact. All parameters were examined under 40 × to 600 × magnification with the OLYMPUS digital microscope DSX-1000 and integrated morphometric stream desktop software (Olympus Hamburg, Germany) [21].

## Statistics

Analyses were performed using Prism 8 software for Mac OS X (GraphPad, La Jolla, CA) running on Apple OS X. The variables were analyzed using the Kolmogorov–Smirnov normality test, and the Mann–Whitney test was used to identify differences between the parameters.

For resonance frequency analysis 30 samples were investigated. A total of 30 samples were evaluated by removal torque. In this investigation 6 implants of 30 were removed. Afterwards, 15 test and 15 control samples were evaluated by histomorphometry and by histological analysis.

A post hoc power analysis was performed with the G*Power software (Heinrich-Heine-Universität, Düsseldorf, Germany) using the post hoc analysis of variance with groups to determine a power of 89% (parameter primary study aim) based on the total sample size of 15 implants per group, using an effect size of 1.23 and an *alpha* of 0.05.

## Results

Of all 80 implants in the 10 pigs, 30 fulfilled the inclusion criteria (no signs of infection, no bone loss in the X-ray or mobility) for the control and test groups. Mainly, 50 implants were excluded due to bone loss. Of the 30 included implants, 15 were randomly selected (test group) and heated with the laser device at a temperature of 50 °C for 1 min.

ISQ values showed no significant differences within each group or between the control and test groups (Fig. 6a). After implant heating with 14 days of follow-up, the mean values were measured at ISQ 79.87 in the test group and at ISQ 73.20 in the control group (Table 1).

**Table 1:** Descriptive statistics of measured distances

Resonance frequency analysis (surgery)		*N*	Mean	standard deviation	Minimum	Maximum
	Test	15	71.87	9.48	51.50	83.50
	Control	15	74.17	9.35	48.00	84.00
Resonance frequency analysis (exposure)	Test	15	72.20	10.84	44.00	86.50
	Control	15	72.03	11.20	52.50	91.00
Resonance frequency analysis (final)	Test	15	79.87	9.95	57.00	95.50
	Control	15	73.20	8.90	57.50	86.00
Bone-to-implant ratio (%)	Test	15	39.76	25.15	2.18	82.78
	Control	15	49.61	34.52	0.00	89.36
Torque-out values (Ncm)	Test	15	88.47	31.12	20.00	0.00
	Control	15	84.00	46.11	110.0	110.0

**Fig. 4:**
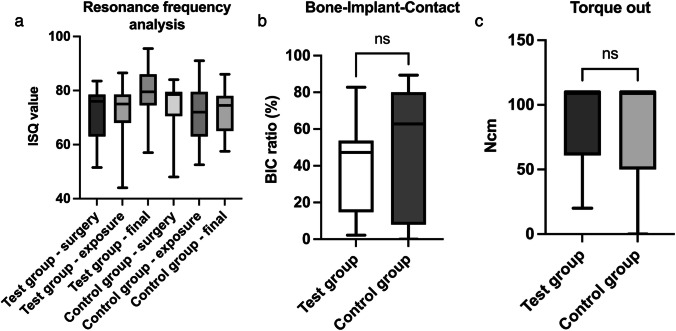
**A **Resonance frequency analysis is shown. Values were measured at three time points for each group. **B **BIC ratio was evaluated at the end of the study and presented in %. **C **Finally, the torque-out measurement was performed for both groups

**Fig. 5:**
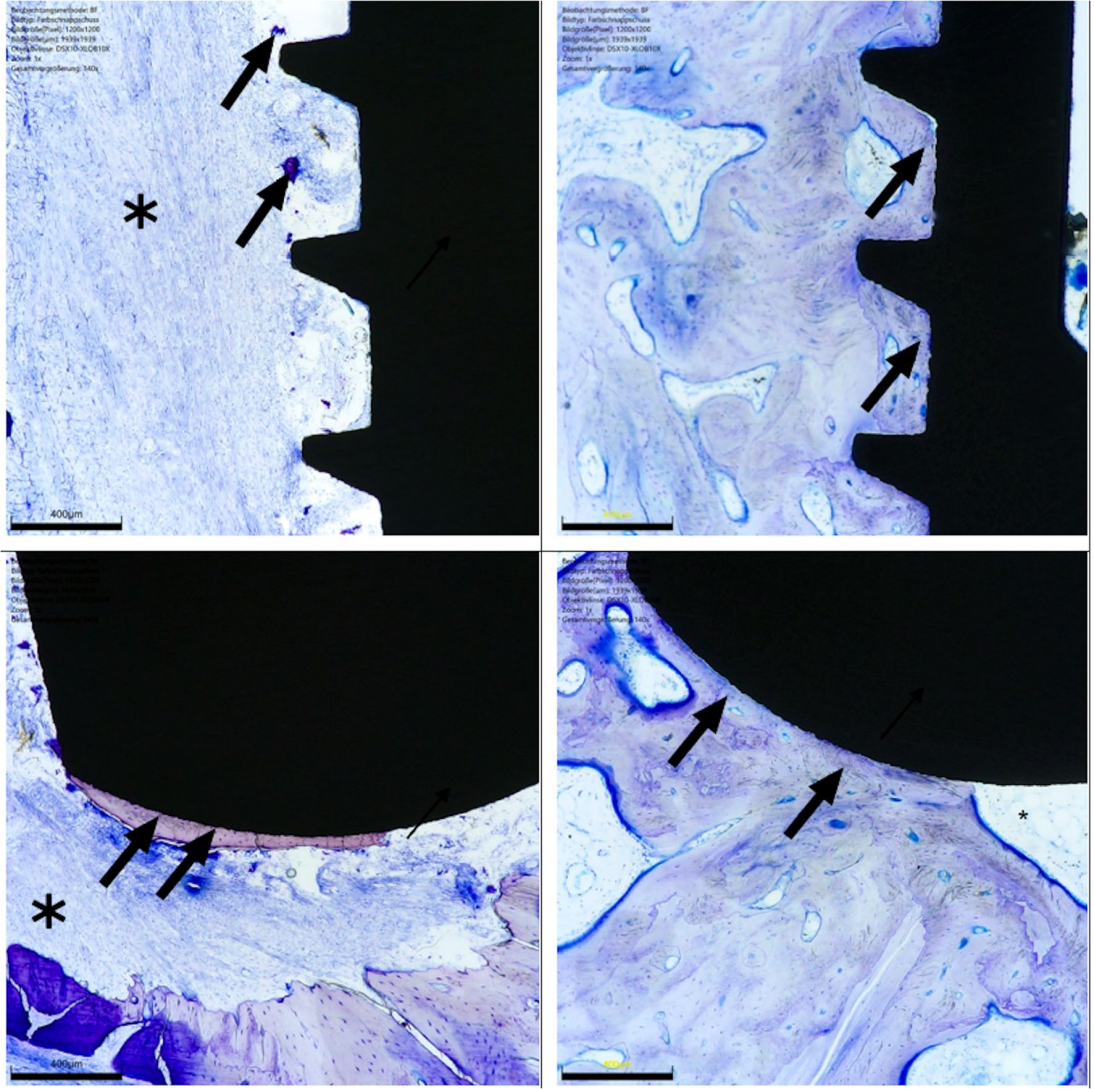
**A **Around an implant of the test group, only a few bone islands with poorly preserved bone material with no implant contact (black arrows). Here, the connective tissue (*) is adjacent mainly in all places. **B **In the overview magnification (digital zoom 1x), the implant of the control group is surrounded by bone tissue in large areas (BIC: 89.36%, bone tissue marked with arrows). **C** In the detail magnification of an implant of the test group (140x), there was only a punctual bone-implant contact (BIC: 5.07%) and small amounts of directly adjacent bone tissue (black arrows) and connective tissue (*) can be seen. **D** Around the implant of the control, the bone tissue (arrows) is directly adjacent to the implant

**Fig. 6:**
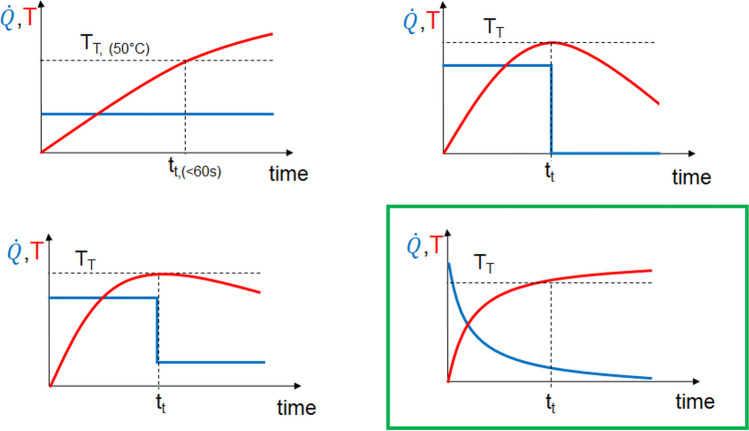
Different time courses of heating and temperature development

At the end of the study, the BIC values of 39.67% in the test group were lower than those of 49.61% in the control group. However, an analysis revealed no significant differences (Fig. 6b). Regarding the torque-out evaluation, again, no significant differences were assessed between the groups (Fig. 4c). The standard deviation of the torque-out measurement in both groups was > 30 Ncm. (Fig. 4c). In the histological examination, differences between test and control groups could be evaluated in several cases. These differences are shown in Fig. 5.

## Discussion

The aim of this study was the evaluation of changes of the implant stability evaluated with the ISQ, the BIC ratio, and the removal torque of implants treated with thermal shock.

In this study authors aimed to initiate only a small layer of necrosis of the bone around the implant to avoid severe bone necrosis. This treatment may reverse the osseointegration of implants and preserve valuable hard tissue. The author’s goal was to heat dental implants in a regulated and calculated manner up to a threshold value. The implant surface should ideally exhibit a uniformly elevated temperature distribution over the whole implant body for thermo-explantation. Atraumatic thermo-explantation could bring benefits to the patient in the future. An uncontrolled heating may lead to severe inflammation and jaw necrosis and would not lead to a better explantation technique [29].

We used a laser device in this study. Heating with laser radiation refers to the use of laser radiation to selectively heat materials. Laser beams can have high energy intensity, making them effective tools for many applications. When laser beams strike a material, the energy from the laser is absorbed by the material and converted into heat. The heating process can be controlled precisely because laser radiation can be directed to a specific area. The laser device has been investigated in a recent study [1]. In the implant body the laser was reflected many times leading to ideal implant heating. Thus, it was not necessary that the laser tip has had contact to the implant itself. The use of laser beams to heat materials has many applications in industry, medicine, and research. In industry, lasers can be used to weld, cut, mark, and solder materials. In medicine, lasers can be applied to treat diseases and diagnostics. Lasers can also be used to study materials and generate plasma in experimental research. It is important to note that care must be taken when using laser beams, as they are very high energy and can cause serious injury if used improperly. Individual case reports described the use of electrosurgical devices that induce local necrosis by heat to remove the implant [17, 18]. In this case, a monopolar electrosurgical device was used that heated the internal thread of the implant for 15 s. This produced localized bone necrosis at the BIC before the implant could be removed one week later using a ratchet at a force < 30 Ncm [17].

To develop an optimal heating scenario for the in vivo large animal model, it first had to be considered that different time courses during heating lead to a completely different temperature development. Figure 6 shows four temporal variants of heating to the target temperature (T_t_ = 50 °C) and at the target time (t_t_ < 60 s) and their temperature response. Based on previous investigations, heating with an exponential approach (Fig. 2, bottom right) was selected for the experiments, since this form of heating minimizes overheating and thus the risk of unwanted thermal damage and bone necrosis.

**Fig. 7:**
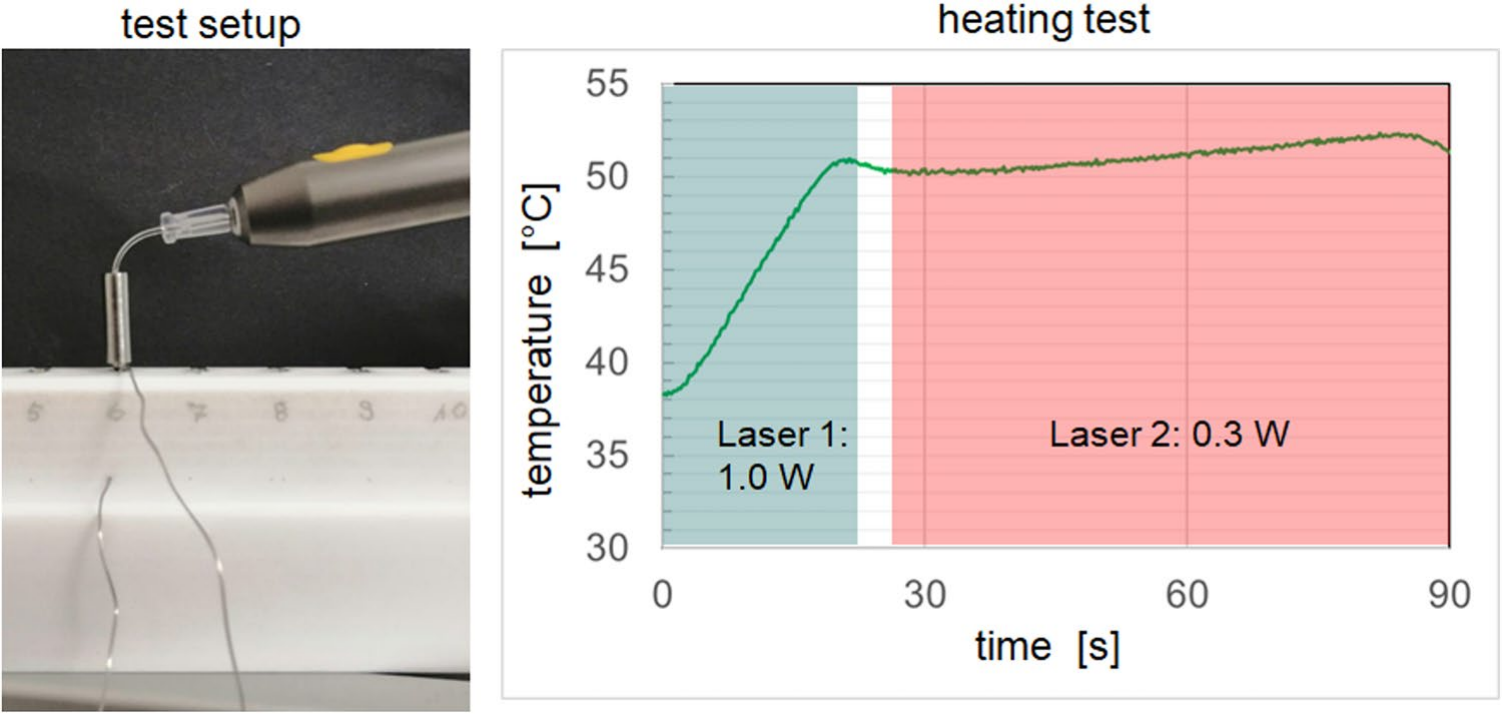
Heating with the dental laser; left: experimental set-up; right: temperature curve with two lasers used in succession

Figure 7 shows the final test set-up for use on the in vivo animal model, with the dental laser and the results of the heating test. For the third surgery, the thermal treatment was carried out by using “2-laser heating” (i.e., two identical lasers with different programmed powers were used consecutively for the thermal treatment; Fig. 7, right side). This was necessary because time-variable programming of laser power was not previously possible in the devices available on the market.

The initial temperature of the implant plays a crucial role in determining the amount of energy required to reach the target temperature during the heating process. Therefore, it was essential to accurately measure the implant’s temperature before heating it.

In anticipation of the investigations, an initial temperature of 37 °C was presumed, which is the average body temperature of a healthy adult human. However, during the implant temperature measurements of the anesthetized animals, it was discovered that their initial temperature was slightly lower, ranging from 34 °C to 36 °C. This discrepancy highlights the importance of conducting precise temperature measurements before any thermal treatment of the implant to ensure that the correct amount of energy is applied to achieve the desired outcome. The deviation in temperature may have been due to several factors, such as individual variations in the animals’ metabolism, anesthesia-induced hypothermia, or the cooling effect of the surgical environment.

It should be noted that the results for removal techniques used have not been consistent or, to date, have only been sporadically described in case reports. To date, there is no procedural protocol that specifies how best to handle implants that require removal. Therefore, a surgical protocol is needed that is reproducible and avoids complications [16].

The authors’ experimental set-up incorporated results previously obtained in another in vivo rat experiments with porcine jaws and implants inserted into the rat tibia [21, 30–32]. These results influenced the choice of tempering method and the temperature and duration of the heating stimulus. To reduce this invasiveness and risk during implant removal, the effect of heat on bone tissue should be considered, which should break the bond between the bone and the implant, even without a mechanically traumatic procedure.

Numerical and experimental investigations were carried out to determine the optimal time course for laser heating of the implant, considering an initial temperature of 37 °C. Based on the results obtained, an empirical time course was developed for the heating power required to achieve the desired temperature:

$${{\text{P}}\left({\text{t}}\right)={\text{P}}}_{\infty }+\left({{\text{P}}}_{0}-{{\text{P}}}_{\infty }\right){{\text{e}}}^{\left(-\frac{{\text{t}}}{\uptau }\right)}$$

with:

$$P_0\left[W\right]=0.13+0.046\cdot{d\cdot l-0.00096\cdot d\cdot l\cdot T}_{In}$$

$$\begin{array}{ccc}P_\infty\left[W\right]=0.66-0.012{\cdot T}_{In}&\text{and}&\tau=100\lbrack s\rbrack\end{array}$$

where P is laser power in W, T_In_ is the initial temperature, d is the implant diameter in mm, l is the implant length in mm, and t is the heating time in s. The developed time course was derived from a combination of numerical simulation and experimental data, which enabled the determination of the required heating power at different time intervals to achieve the desired temperature. This empirical time course provides a practical guide for the application of laser heating, as it accounts for variations in the geometrical parameters of the implant, laser power, and initial temperature.

The empirical time course for heating with the two lasers is illustrated in Fig. 3. Specifically, the figure shows the time required to achieve the desired temperature. It is worth noting that the empirical time course may require modification for implants with large deviations in temperature, geometries, or material properties. However, it provides a useful starting point for the application of laser heating and may help optimize the thermal treatment of implants for enhanced clinical outcomes.

The aim of the present study was to ensure the most atraumatic removal possible, which would have an advantage over the implant removal methods currently described. Some techniques rely on the use of drills, such as trephine drills. The objective is to deliberately weaken the bone and reduce the bond between the bone and the implant until the implant can be removed [33–35]. This technique deliberately accepts the loss of bone substance and requires conscientious surgical planning, with precise knowledge of the course of the vessels. Fractures of the mandible and osteomyelitis may occur, as observed in some case reports [36]. The counter torque ratchet technique (CTRT), which is the most frequently applied, uses a ratchet to rotate the implant out of the bone in a counterclockwise direction [37–40]. This procedure applies a force that is as high as 500 Ncm [16]. According to Anitua et al., [37] this procedure was successful in 87.7% of cases. In the case of incorrectly positioned, fractured implants, or those that still show a high BIC ratio despite peri-implantitis and require more than 200 Ncm for removal, explantation using CTRT is not directly feasible. In this case, the BIC was first reduced using trephine drills. Excessive force may result in fracture of implant [13] or bone [41]. Furthermore, the use of the trephine drill can cause deep bone defects that make reimplantation difficult.

Our study could not prove the concept of thermal implant removal on osseointegrated implants. This could be due to several factors. On the one hand, bone is a multivariate structure that can differ greatly from creature to creature, even within a single breed. Thus, a high bone density with lower peri-implant blood flow influences the target temperature at the implant surface, since the heat is less removed. On the other hand, the average body temperature determined can also differ individually, depending on the day and the individual’s health. These factors therefore influence the final surface temperature of the heated implant and might change the intended temperature. A critical reflection of the implant success rate showed that a large number of implants had to be excluded due to bone loss. A possible cause could be found in the animal experiment due to heavy masticatory stress. Future studies should test a longer follow-up interval. It should not be neglected that the clinical relevance of such a procedure should not take too long.

## Conclusion

In this animal study, with a temperature of 50 °C for 1 min, the de-integration of implants was not successful. At 50 °C, changes in the BIC values were noticeably smaller; however, these differences were not significant. Future studies should evaluate the same procedures at either a higher temperature or longer intervals.

## Data Availability

The datasets used and/or analyzed during the present study are available from the corresponding author on reasonable request.
